# Highly individual- and tissue-specific expression of glycoprotein group A and B blood antigens in the human kidney and liver

**DOI:** 10.1186/s12865-021-00456-2

**Published:** 2021-10-01

**Authors:** Xianding Wang, Fan Zhang, Yamei Jiang, Zilin Xu, Xiaobing Feng, Linde Li, Yu Fan, Turun Song, Yunying Shi, Zhongli Huang, Tao Lin

**Affiliations:** 1 Department of Urology/Institute of Urology, West China Hospital, Sichuan University, Number 37, Guoxue Alley, Chengdu, 610041 Sichuan China; 2 Organ Transplantation Center, West China Hospital, Sichuan University, Chengdu, Sichuan China; 3 Department of Urology/Shanghai Key Laboratory of Organ Transplantation, Zhongshan Hospital, Fudan University, Shanghai, China; 4 West China School of Clinical Medicine, Sichuan University, Chengdu, Sichuan China; 5 Department of Nephrology, West China Hospital, Sichuan University, Chengdu, Sichuan China

**Keywords:** ABO incompatible transplantation, Blood group antigen, Kidney, Liver, Western blot

## Abstract

**Background:**

Currently, research on the quantitative distribution of ABO antigens in different organs and tissues remains limited. We aimed to examine the individual characteristics of blood group glycoprotein A and B antigen expression in human kidneys and livers.

**Methods:**

We obtained human samples, including the renal artery, renal vein, renal tissue, hepatic artery, hepatic vein, portal vein, and hepatic tissue, from 24 deceased organ transplant donors. The expression of the blood group antigens glycoprotein A and B was analysed and compared by Western blotting.

**Results:**

There was no significant difference in the expression between blood group glycoprotein A and B antigens at any of the seven sites (*p* > 0.05). The expression of both A and B antigens was highest in renal tissue and the portal vein and was lowest in the renal artery. A large difference in glycoprotein antigen expression was observed among various donors or different regions of the same individual. Univariate analysis revealed that glycoprotein A/B antigens were affected by the age and sex of donors and were significantly higher in males and in young people.

**Conclusions:**

Our study found that blood group glycoprotein antigen expression showed certain trends and distinct distribution in the kidney, liver, and vessels among individuals and in different regions of the same individual, which may explain the different clinical outcomes of patients who received ABO-incompatible transplantation.

## Introduction

The transplantation of ABO-incompatible (ABOi) donor allografts into recipients with naturally occurring anti-A or B antibodies may sometimes result in antibody-mediated rejection (AMR); this process is initiated by antibodies binding to A or B antigens present on the vascular endothelium within the graft and subsequent complement activation [1]. Thus, pre-existing anti-donor ABO antibodies in recipient blood must be removed before ABOi transplantation, and antibody rebound must be prevented after transplantation [2, 3]. Currently, clinical preconditioning protocols exclusively consider the anti-donor ABO antibody titre, which must be reduced to the “safe” range (e.g., ≤ 1:16 in most transplant centres) on the transplant day [4–6]. Performing organ transplantation in ABO-incompatible cases continues to pose a significant challenge [7]. Even when the ABO antibody titre was “safe” on the day of transplantation, some ABOi recipients still experienced AMR or even hyper-acute rejection [8, 9]. In contrast, some ABOi allografts survived without AMR when the posttransplant ABO antibody titre gradually increased to the preoperative level [10, 11]. It is unclear why these two contrasting events occur.

To date, most studies have focused on the anti-donor ABO antibody titre of recipients, and very little is known about the influence of the donor expression pattern of A/B antigens on posttransplant AMR incidence. It is hypothesized that, in addition to ABO antibodies, the onset and absence of AMR in these ABOi recipients may also be attributed to the individual pattern of distribution, subtype, and levels of ABO blood group antigens within the allograft. Several previous studies have described the expression pattern of organ- surface A/B antigens. On the vascular endothelial cells of the human heart, only type II A/B antigens were expressed, and type III/IV structures were not detected. Conversely, in children who received ABOi heart transplants, ABO antibodies specific for type II antigens—the only A/B antigen subtypes expressed in heart tissue—were absent, demonstrating the high specificity of B cell tolerance to donor blood group antigens [12–14]. In the human renal vascular bed, a previous study reported that there were three different A antigen expression patterns (major, minor, and minimal staining distribution), while all kidneys showed a B antigen pattern that was similar to the major pattern of A antigen but was slightly weaker [15]. These expression profiles have important implications that should be considered in clinical settings of ABOi transplantation.

However, these previous studies are limited because A/B antigen quantification was performed with only immunohistochemistry (IHC), which may be particularly difficult to interpret. A/B antigens are expressed in tissues as portions of glycolipids and glycoproteins. Highly individual- and tissue-specific expression of glycolipid group A and B blood antigens was found in previous studies [16], but no studies have used Western blot analysis to quantify glycoprotein A/B antigens; previous studies used tissue samples in which red blood cells (RBCs) were present at high levels, making it impossible to distinguish between erythrocytic and tissue sources of ABO blood group antigen expression with Western blotting. Thus, the purpose of this study was to evaluate the hypothesis that the quantitative glycoprotein A/B antigen expression in RBC-free human kidneys and livers (the two most common organs used for ABOi transplantation) varies greatly among individuals as well as among different sites in an individual based on Western blotting.

## Materials and methods

### Tissue samples

This study protocol was reviewed and approved by the Biomedical Ethics Committee, West China Hospital (No. 2019SHEN282). All organ donation cases were conducted according to the protocols for China Categories I, II, and III donors [17]. All kidney [renal artery, renal vein, renal tissue (by a 14-gauge BARD MAX-CORE disposable core biopsy instrument)] and liver [hepatic artery, hepatic vein, portal vein, hepatic tissue (by wedge resection)] samples were obtained from deceased organ transplant donors. The donor kidney and liver were perfused before and immediately after being removed surgically (to remove residual red blood cells) and preserved in an RBC-free histidine-tryptophan-ketoglutarate solution. For blood groups that were positive for antigen A, namely, groups A and AB, further tests were performed with anti-A1 lectin to determine the subtype of the blood A antigen (A1 or A2).

### Eligibility criteria

The eligibility criteria for deceased donors were as follows: (1) written consent obtained from the next of kin, (2) 18–65 years old, (3) ABO blood group, (4) organ donation after cardiac or brain death, (5) normal terminal serum creatinine [< 110 µmol/l (female) or < 140 µmol/l (male)], (6) normal terminal serum alanine aminotransferase [< 40 IU/L (female) or < 50 IU/L (male)], and (7) the donated kidneys and livers were used for subsequent ABO-compatible organ transplantation and had normal graft function.

Exclusion criteria included (1) acute kidney or liver injury before donation, (2) a medical history of chronic kidney disease or hepatitis B or C virus infection, (3) active infection needing antibiotic treatment, and (4) renal or liver parenchyma damage or lithiasis revealed by ultrasound.

### Western blot analysis

Total protein was extracted from tissue samples, the tissues were homogenized, and centrifuged at 4 °C. The supernatants were used for Western blot assays. Proteins were separated by 10% SDS-PAGE and then transferred onto PVDF membranes (Amersham, GE Healthcare, Chicago, IL). After being blocked with 5% nonfat milk, membranes were incubated with an anti-blood group A antibody (1:1000, BIOSCOT, Merck Millipore) at 4 °C overnight, with a mouse monoclonal anti-β-actin antibody (1:10,000, ab8245) used as the internal reference, followed by a subsequent incubation with a goat anti-rabbit secondary antibody (1:8000, Santa Cruz Biotechnology, Santa Cruz, CA, USA). The visualization of the protein bands was performed with enhanced chemiluminescence kits (Santa Cruz Biotechnology). The band intensity was quantified using Image Lab 6.0.1 Software (Bio-Rad Laboratories, Inc). The relative expression of blood group antibodies is represented by the ratio of the band intensity between the experimental band and internal reference.

#### H&E and immunohistochemical staining

Specimens from human kidneys and livers were fixed in formalin and embedded in paraffin. For H&E staining, 4-µm slices were stained with H&E for histological examination. For immunohistochemistry of blood group A antigens, samples were rehydrated, washed with TBS, and blocked with TBS containing 10% FCS and 1% BSA, followed by incubation for 16 h in TBS containing 1% BSA and primary antibodies for blood group A or B antigens. Tissue sections were washed twice with TBS, and then HRP-conjugated anti-IgG secondary antibodies were added to TBS. After washing twice, sections were counterstained with Mayer’s haematoxylin, and images were analysed with a Nikon eclipse 90i digital microscope.

### Statistical analysis

Experimental data are presented as the mean ± standard deviation (sd) or median (range). Mean values of the groups with normally distributed data were compared using Student's t-tests or ANOVA, whereas Wilcoxon rank-sum tests and Friedman M tests were used for comparisons of non-normally distributed data. All statistical analyses were conducted using R (version 3.4.4), and a *P*-value below 0.05 was considered significant.

## Results

In this study, we obtained 168 tissue samples from 24 deceased organ donors, including 13 (54.2%) donors in group A, 2 (8.3%) donors in group AB, 6 (25%) donors in group B, and 3 (12.5%) donors in group O; there were 15 (62.5%) males and 9 (37.5%) females, with a mean age of 50.29 ± 12.32 years (Table 1). All the donors in groups A and AB belonged to the A1 subtype. The mean height was 1.64 ± 0.08 m, and the mean weight was 62.67 ± 12.76 kg, with a mean body mass index (BMI) of 23.04 ± 4.00 kg/m^2^. The deceased donors died of a variety of causes: head trauma (n = 13, 54.2%), stroke (n = 10, 41.7%), and central nervous system tumours (n = 1, 4.2%).

**Table 1:** Clinical parameters of included deceased organ transplant donors

Feature	N = 24
*Blood type [n (%)]*	
A	13 (54.2%)
B	6 (25%)
AB	2 (8.3%)
O	3 (12.5%)
*Gender [n (%)]*	
Male	15 (62.5%)
Female	9 (37.5%)
Age (years) [mean ± sd]	50.29 ± 12.32
Height (meters) [mean ± sd]	1.64 ± 0.08
Weight (kilograms) [mean ± sd]	62.67 ± 12.76
BMI (kg/m2) [mean ± sd]	23.04 ± 4.00
*Cause of death [n (%)]*	
Head trauma	13 (54.2%)
Stroke	10 (41.7%)
Central nervous system tumor	1 (4.2%)

*BMI* Body Mass Index

To eliminate false-positive errors, specimens from blood group O were incubated with anti-blood group A and B antibodies, and A/B antigens were not detected in type O organs. In addition, B antigen expression was not detected in blood group A specimens, and vice versa.

**Fig. 1:**
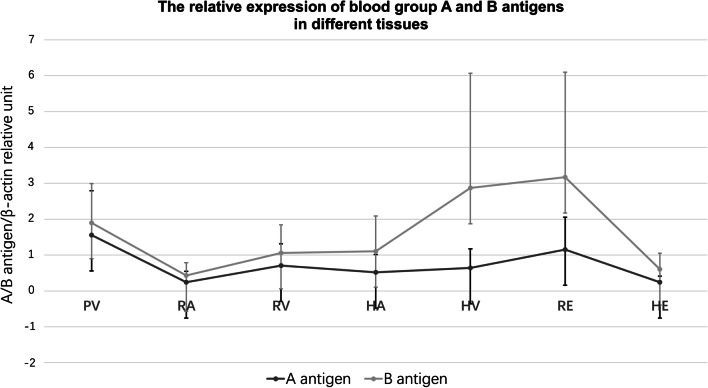
Western blot results showing the relative expression of blood group A and B antigens in different tissues. The tissue samples included 88 samples from 15 blood group A donors and 49 samples from 8 blood group B donors. Research data are presented as the mean ± standard deviation. PV: portal vein, RA: renal artery; RV: renal vein; HA: hepatic artery; HV: hepatic vein; Renal: kidney tissue; Hepatica: liver tissue

**Fig. 2:**
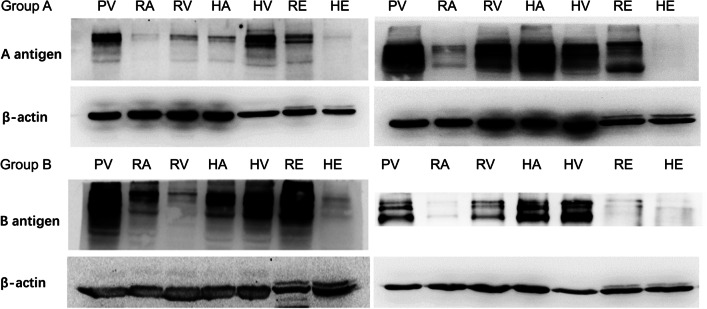
Western blot analysis of blood group antigens from two antigen A donors and two antigen B donors. PV: portal vein; RA: renal artery; RV: renal vein; HA: hepatic artery; HV: hepatic vein; RE: kidney tissue; HE: liver tissue

The glycoprotein A and B antigen intensities at the seven sites are displayed in Fig. 1. Western blot analysis of blood group antigens from partial samples is shown in Fig. 2. The mean intensities of A antigen among the 13 type A donors and 2 type AB donors were 0.25 ± 0.30 in the renal artery, 0.25 ± 0.17 in hepatic tissue, 0.52 ± 0.50 in the hepatic artery, 0.64 ± 0.53 in the hepatic vein, 0.71 ± 0.61 in the renal vein, 1.16 ± 0.90 in renal tissue, and 1.56 ± 1.23 in the portal vein, in increasing order. The mean intensities of B antigen among the 6 type B donors and 2 type AB donors were 0.43 ± 0.36 in the renal artery, 0.61 ± 0.44 in hepatic tissue, 1.06 ± 0.78 in the renal vein, 1.10 ± 0.98 in the hepatic artery, 1.89 ± 1.10 in the portal vein, 2.87 ± 3.20 in the hepatic vein, and 3.20 ± 2.92 in renal tissue, in increasing order. There was a 6.24-fold difference in the mean glycoprotein A antigen intensity between the lowest- and highest-intensity sites and a corresponding 7.44-fold difference in the glycoprotein B antigen intensity.

Much wider individual differences in blood group antigen intensities were observed at the same site of various donors as well as at different sites of the same donor. The Friedman M test was then used and indicated that there were differences in the distribution among the seven sites of the glycoprotein A antigen group (*p* < 0.001), but there was not a significant difference in the glycoprotein B antigen group (*p* = 0.544). Individual differences were significantly different in both the glycoprotein A antigen group (*p* = 0.004) and B antigen group (*p* = 0.002). The fold difference between the highest and lowest A antigen intensities at the same site among the 13 type A donors and 2 type AB donors was 98 (0.98/0.01) in the renal artery, 213 (2.13/0.01) in the renal vein, 54.2 (3.25/0.06) in renal tissue, 81.5 (1.63/0.02) in the hepatic artery, 10.2 (1.53/0.06) in the hepatic vein, 11.4 (5.00/0.44) in the portal vein, and 10.2 (0.51/0.05) in hepatic tissue. The fold difference between the highest and lowest B antigen intensities at the same site among the 6 type B donors and 2 type AB donors was 56.5 (1.13/0.02) in the renal artery, 36.2 (2.17/0.06) in the renal vein, 63.3 (8.23/0.13) in renal tissue, 21.7 (2.82/0.13) in the hepatic artery, 48.9 (10.26/0.21) in the hepatic vein, 9.88 (3.26/0.33) in the portal vein, and 8.7 (1.31/0.15) in hepatic tissue. The median fold difference between the highest and lowest A antigen intensity at various sites in the same donor was 11.9 (range 4.15–317), while the median fold difference between the highest and lowest B antigen intensity at various sites in the same donor was 20.1 (range 4.03–70.75).

Univariate analysis revealed that blood group glycoprotein antigen expression in males was significantly higher than that in females (*p* = 0.048) in both the A and B groups (Table 2). Furthermore, we found that glycoprotein antigen expression was higher in young people (*p* = 0.038). No association was observed between antigen expression intensity and the BMI or transfusion history of the donor (Table 2).

**Table 2:** Univariate analysis of clinical features related to blood group antigen expressions

Feature	*P* value							
	PV	RA	RV	HA	HV	RE	HE	Total
*Sex*								
A	0.567	**0.045**	**0.003**	**0.001**	0.365	0.300	–	**0.008**
B	–	0.738	0.749	0.832	0.958	0.718	0.543	0.739
A&B	0.920	0.238	**0.027**	0.130	0.598	0.295	0.568	**0.048**
*Transfusion*								
A	**0.090**	0.168	0.475	0.262	0.187	0.580	0.848	0.675
B	**0.036**	0.990	0.698	0.471	0.321	0.934	0.747	0.367
A&B	**0.005**	0.237	0.719	0.252	0.227	0.783	0.872	0.699
*Age*								
A	0.648	0.382	0.309	0.178	0.678	0.646	**0.050**	0.459
B	0.890	0.504	**0.042**	**0.009**	0.446	0.165	–	**0.029**
A&B	0.690	0.908	0.121	0.065	0.464	0.204	0.809	**0.038**
*BMI*								
A	0.722	0.380	0.351	0.850	0.714	0.885	–	0.874
B	–	0.124	0.133	**0.006**	0.266	0.177	0.255	**0.009**
A&B	0.926	0.940	0.153	0.208	0.398	0.143	0.350	0.054

Patients were grouped and then compared by male/female, transfusion history/no transfusion history, cut-off value of median age, or cut-off value of median BMI value. *P* < 0.05 was regarded significantly different and marked in bold

*PV* portal vein; *RA* renal artery; *RV* renal vein; *HA* hepatic artery; *HV* hepatic vein; *RE* kidney tissue; *HE* liver tissue

**Fig. 3:**
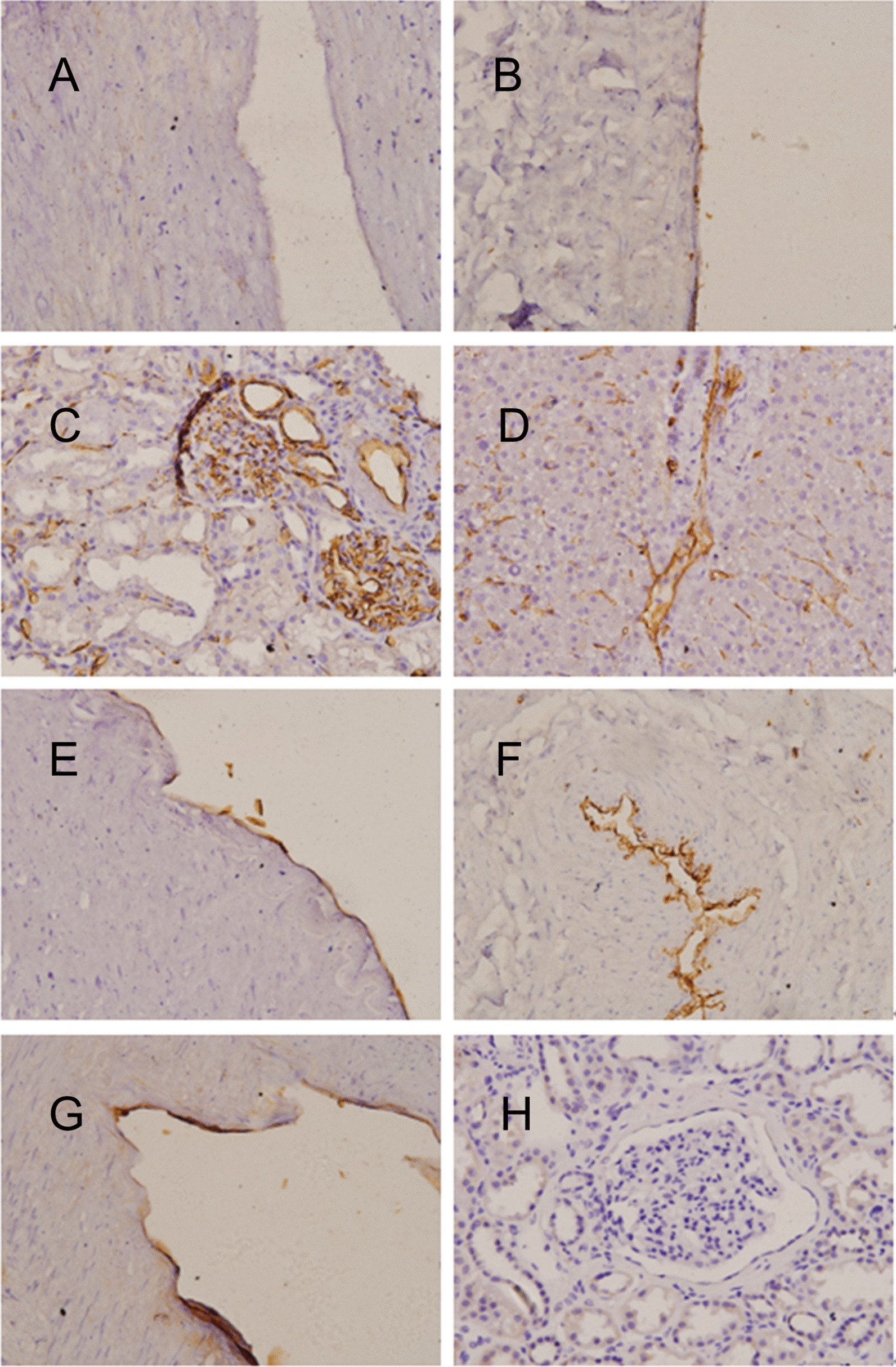
Immunohistochemical staining of the renal artery (**A**), renal vein (**B**), portal vein (**C**), hepatic tissue (**D**), hepatic artery (**E**), hepatic vein (**F**), renal tissue (**G**) of an A antigen donor and the renal tissue (**H**) of a blood type O donor. RA, renal artery; RV, renal vein; RE, renal tissue; HE, hepatic tissue; HA, hepatic artery; HV, hepatic vein; PV, portal vein

In all IHC sections, blood group antigen is shown in red, and nuclei are represented in blue. The intensity and distribution of the A antigen showed distinctly different patterns in different tissues (Fig. 3). The renal tissue was most strongly stained, and positivity was mainly found in the glomerular and tubular capillaries. In the renal artery and renal vein, the immunostaining was relatively weaker, which was most obvious in the renal artery. In the renal vein, portal vein, hepatic artery, and hepatic vein, positive staining was mainly seen in the vascular bed, where the endothelium was stained, whereas in liver tissue, positive staining was mainly found in the venae centrales hepatis and hepatic sinusoid. The intensity and distribution of blood group antigens showed distinctly different patterns in different tissues in both WB and IHC results.

## Discussion

ABO blood group antigens are carbohydrate chains expressed on the cell membrane of RBCs. The H antigen is the only ABO structure present in blood type O, and A/B blood group antigens contain the H antigen as a common precursor [18]. Blood group A individuals express A antigens on their RBCs. Similarly, blood group B individuals express B antigens, group O individuals express neither A nor B antigens, and group AB individuals express both [19]. In addition to RBCs, ABO blood group antigens are also distributed on lymphocytes, platelets, and most epithelial and endothelial cells. The targets of blood group antibodies in ABOi transplantation are endothelial cells, but the antigen expression patterns of endothelial cells are not faithfully modelled by RBCs. Currently, there is little known about the endothelial expression of blood group antigens in human organs. Vascularized allografts are the targets of blood group antibodies, which, upon binding to endothelial cell surfaces, activate the complement system, which in turn mediates AMR [20].

The amount of antigen displayed on a specific organ may be of particular importance in ABOi transplantation. Gehrie et al. performed IHC staining of blood group A antigen in tissue blocks of 18 blood type A cadaveric hearts obtained at autopsy [12]. Light microscopic examination found that the endothelium stained with a low intensity in 4 (22%) myocardial samples, an intermediate intensity in 5 (28%) samples, and a high intensity in 9 (50%) samples. The digital analysis of IHC staining intensity and percentage of the total surface area of immunoreactivity revealed a 20-fold difference between the lowest and highest expressing specimens, and immunoreactivity was positively associated with patient age (*p* = 0.004). Breimer et al. examined the A/B antigen expression of renal biopsy samples by IHC [15]. In the human renal vascular bed, three different A antigen expression patterns with a major, minor, and minimal staining distribution were observed. The major pattern showed intense A antigen expression in the endothelium of arteries, glomerular/peritubular capillaries, and veins. A minor pattern showed overall weaker antigen expression, whereas minimal had faint staining of peritubular capillaries only. In all cases, the distal tubular epithelium was focally stained, whereas proximal tubules were negative. The secretor gene did not influence renal A antigen expression. All B kidneys showed a B antigen pattern slightly weaker but similar to the major pattern of the A antigen.

However, the major limitation of these previous studies was that the IHC analysis of blood groups may be subject to intra- and interobserver variability. Meanwhile, other techniques for blood group antigen detection—such as Western blotting—could have been affected by contaminating RBCs that are ubiquitous in autopsy and biopsy specimens [20]. RBCs made it difficult to distinguish between erythrocytic and tissue sources of blood group antigen expression. The existence of RBCs may lead to an overestimation of the relative expression in WB and IHC results. Thus, autopsy samples cannot be used to accurately quantify blood group antigens. Our study used RBC-free histidine-tryptophan-ketoglutarate solution to perfuse donor kidneys and livers to eliminate residual RBCs. Thus, there should have been few, if any, residual RBCs in our tissue samples. A previous study found highly individual- and tissue-specific expression of glycolipid group A and B blood antigens, but no studies have analysed and compared glycoprotein A/B antigens. Thus, we detected the relative expression of blood group glycoprotein antigens in kidney and liver tissues with Western blotting.

Our study found that the mean intensity of glycoprotein A and B antigen was generally similar (*p* > 0.05). Furthermore, kidney tissue and the portal vein were the two sites with the highest average intensity, and the renal artery was the site with the lowest intensity. It was not surprising that the injury in renal tissue would be more severe than that in the main renal vessels when blood group AMR occurred. The high level of blood group antigens in the portal vein may be related to the high incidence of portal vein thrombosis and portal vein stenosis after liver transplantation. Additionally, the comparison of the intensity in kidney versus liver tissues indicated that there was a much higher expression level of glycoprotein antigen in renal tissue than in liver tissue from the same donor, which might partially explain why ABOi kidney transplantation requires more aggressive pre-transplant preconditioning than ABOi liver transplantation and why the incidence of AMR in ABOi kidney transplantation was more higher than that in ABOi liver transplantation. We also found a wide diversity of blood group glycoprotein antigen intensities at the same site from a cohort of different deceased donors as well as at different sites from the same donor. These observations may explain why even when the ABO antibody titre was “safe” on the transplant day, two allografts in our case series of 48 ABOi recipients exhibited immediate failure and pathology consistent with hyperacute rejection [21].

Thus, pre-transplant evaluation of the risk and severity of ABO blood group AMR may be important when performing ABOi kidney transplantation and may allow individualized preconditioning regimens based on A/B antigen intensity and diversity. For example, when the organ tissues and main vessels in a donor express a high level of blood group antigens, the risk of AMR in the corresponding ABOi recipients may be likely to be higher, and such recipients may need more intense preconditioning to decrease the ABO antibody titre to an even lower level [22]. Worldwide, there are currently no safe, non-invasive, and reliable methods that can be used to quantify and inhibit ABO blood group antigen expression in donor organs. As a result, the preconditioning regimen in ABOi organ transplantation has been limited to decreasing the blood group antibody titre in the recipients. Our research revealed the prominent individual diversity of glycoprotein intensity for the ABO antigen in different organs and tissues by Western blotting. These results are useful for the further study of risk stratification and preconditioning regimens based on donor blood group antigen expression levels in ABOi organ transplantation. This study also provided important experimental datato further illustrate the mechanism of ABOi AMR.

Two weeks after ABOi transplantation, an antigen–antibody reaction does not occur, and the ABOi allografts function normally, despite the presence of A/B antigens on the graft and the existence of antibodies directed against the corresponding antigens in the blood of the recipient [23–25]. This type of tolerance is referred to as “accommodation”, and its underlying mechanisms remain to be elucidated. Accommodation may be due to (1) a change in the antigen, leading to decreased antibody binding; (2) a change in antibodies, reducing their cytotoxicity; or iii) a change in the graft, enabling it to resist the injury mediated by the host immune system [16, 26, 27]. During clinical transplantation accommodation, these processes can manifest as the inhibition of the activity of glycosyltransferase, resulting in decreased antigenic immunogenicity. On the other hand, antigen downregulation might underlie the process of accommodation in which a graft survives and functions despite the presence of anti-donor antibodies in the circulation of the recipient. Ulfvin et al. analysed glycolipids extracted from ABO-incompatible allografts that had become accommodated and found the accommodated grafts had reduced levels of antigen, suggesting that antigen down-modulation might play a role in the long-term function of the organs [28].

This study had several limitations. First, the statistical power may have been limited due to the relatively small sample size. Second, all individuals in the A and AB blood groups had A1 subtypes because the frequency of A2 in the Chinese population is < 1%, and no A2 individuals were included in this study. Finally, all livers and kidneys from the deceased donors underwent subsequent ABO-compatible but not ABOi organ transplantation. Thus, the association between A/B expression patterns and blood group AMR remains to be elucidated. A large prospective clinical trial is needed to confirm our current findings and explore the association of A/B expression patterns with AMR and graft function in ABOi organ transplant recipients. Further studies might determine whether there is a threshold of the expression of blood group antigen that is likely to trigger AMR.

## Conclusions

Our study revealed substantial inter-individual variations in blood group A and B antigens between human livers and kidneys. These variations may explain differences in the incidence and clinical severity of AMR in ABOi kidney/liver transplantations and could theoretically be used to risk-stratify ABOi organ transplant procedures. Future studies may consider non-invasive detection methods for the intensity of blood group antigens in a specific donor organ, which could more accurately assess the risk of AMR in ABOi transplantation recipients and help to select more precise desensitization regimens to perform ABOi organ transplantation safely.

## Data Availability

The datasets used and/or analysed during the current study are available from the corresponding author on reasonable request.

## Abbreviations

ABOi: ABO-incompatible
AMR: Antibody-mediated rejection
IHC: Immunohistochemistry
BMI: Body mass index
PV: Portal vein
RA: Renal artery
RV: Renal vein
HA: Hepatic artery
HV: Hepatic vein
RE: Kidney tissue
HE: Liver tissue
